# Indoor and Outdoor Air Microbial Contamination During Different Reconstruction Methods of Historic Buildings

**DOI:** 10.3390/pathogens13121048

**Published:** 2024-11-29

**Authors:** Anett Lippai, Ádám Leelőssy, Donát Magyar

**Affiliations:** 1 Biokör Technological and Environmental Protection Ltd., 1089 Budapest, Hungary; 2 Department of Meteorology, Institute of Geography and Earth Sciences, ELTE Eötvös Loránd University, 1053 Budapest, Hungary; adam.leelossy@ttk.elte.hu; 3 National Public Health and Pharmaceutical Centre, 1097 Budapest, Hungary; magyar.donat@gmail.com

**Keywords:** air microbiology, reconstruction, fungi, indoor and outdoor air pollution, disinfection, dispersion model

## Abstract

The quality of indoor air is dependent on a number of factors, including the presence of microorganisms that colonize the building materials. The potential for health risks associated with microbial contamination is a significant concern during the renovation of buildings. The aim of this study was to assess the impact of two reconstruction methods for historic buildings on air quality. The two reconstruction procedures were facadism, which preserves only the façade, demolishing the rest of the building and constructing a new building, and complete reconstruction, which involves internal renovation with a less intensive demolition. A total of 70 + 70 air samples, as well as surface and dust samples, were collected throughout the course of the reconstruction of the two buildings. In the case of facadism, total colony counts were found to be 2–4 times higher indoors than outdoors, even at the initial stage of the works. High concentrations of *Aspergillus* and *Penicillium* spp. were detected. During the less intensive reconstruction, the total colony count in the indoor air samples was initially lower at almost every sampling point than at the outdoor levels. With regard to fungi, *Penicillium* species were initially present at lower conidia concentrations, followed by *Aspergillus* species over time. In both buildings, elevated concentrations of airborne fungi were detected during the main reconstruction period. The fungal genera found in the indoor air were also detected on surfaces and in dust samples. Outdoor air samples collected from the vicinity of the buildings revealed elevated fungal counts at multiple sampling points, particularly in the case of facadism. Disinfection with dry fogging was implemented twice throughout the entire interior of the buildings. Following the first disinfection process, there was no notable decrease in colony-forming unit (CFU) counts in either building. However, the second disinfection resulted in a reduction in microbial concentration in the air. Our study confirms that the renovation of historical buildings can result in an elevated prevalence of fungal bioaerosols, which can be harmful to occupants. While the impact of the reconstruction remained within the range of urban background variability at distant (>1 km) locations, it caused local microbial contamination, often exceeding the detection limit in near-site samples.

## 1. Introduction

The air quality of indoor environments is of significant consequence to our health, well-being, and productivity. It is estimated that people typically spend 80–90% of their time in indoor environments. This has led to a growing interest in indoor microbiological studies in recent years [1]. Indoor air quality is influenced by chemical, physical, and biological factors. Concerning the biological factors, poor indoor air quality is often caused by bacteria, molds, and yeasts, which can emit spores and different metabolites, which increase the risk of respiratory diseases such as allergies, asthma, irritation, and infections [2].

Aged building materials are a characteristic feature of historical buildings, often exposed to the effects of water access and subsequent biodegradation by fungi and bacteria. Some of these historical buildings hold high cultural value and must be preserved for future generations. Their preservation, renovation, or reconstruction involves intensive handling of aged building materials colonized by microorganisms [3]. Therefore, microbial risks must be considered during the reconstruction procedures of old buildings.

Two types of reconstruction procedures are known: facadism and complete reconstruction [4,5]. The general definition of facadism is the preservation of an existing historical façade and its incorporation into a new build element. In this case, the building is renovated by maintaining only the facades, but the rest of the building is demolished, and a new structure is built. Facadism, which first emerged in the UK and Italy in 1939, represents an intermediate approach between the conservation and development of buildings. Nowadays, facadism is a growing urban trend and a practical way to preserve the historic character of a building [4,5]. Complete reconstruction is also a widely used approach, particularly for historic buildings and architectural monuments. Complete reconstruction involves internal renovation with minimal demolition and is applied when the preservation of the existing building is essential [6].

The international literature contains several studies focusing on indoor air quality: investigations of libraries and university rooms [1,2], hospitals [7,8,9], wooden churches [3], and office buildings [10]. With regard to the impact of reconstruction works on indoor air quality, the available information is limited. There are only a few studies in this area, most of them focusing on microbiological investigations during the demolition or renovation of hospitals [11,12,13,14,15,16]. Such studies demonstrated increased spore emissions during reconstruction works, which are associated with an increased incidence of fungal infections in hospitalized people. Due to high health risks, management and disinfection of indoor microbial air quality are of outmost importance. Although the reconstruction of historical buildings is frequently performed in densely populated urban areas, particularly in city centers, we were unable to find any studies dedicated to its potential impact on human health. The majority of studies focus on the fungal contamination of the indoor air during demolition, but the environment of the renovated building is typically overlooked. Our working hypothesis was that the various phases and techniques used in reconstruction works on historical buildings have a major influence on the concentration of airborne fungi.

This study aims to 1. determine the concentrations of airborne fungi in two historical buildings applying different reconstruction methods; 2. compare the impact of the two different reconstruction methods on indoor air quality; and 3. investigate the impact of reconstruction works on outdoor air quality.

## 2. Materials and Methods

Air samples were collected between May 2022 and February 2023 in Budapest, Hungary, during the initial phase of building renovations. Two historical buildings were investigated in the center of the town, situated in close proximity to each other. One building underwent a complete reconstruction (CRB), while facadism was applied to the other (FB). The buildings are situated in an urban environment, near the Danube River among historical buildings. Both sampled buildings were constructed at the end of the 1880s in the Neo-Renaissance/Neo-Baroque style; the CRB was reconstructed in the 1940s. The floor areas of the two buildings were similar (CRB: 7000 m^2^; FB: 7650 m^2^). The CRB building had a ground floor, 1/2^th^ floor, 5 floors, and a cellar, while the FB building had an entresol, ground floor, and 2 floors. Samples were collected from all floors and the cellar. A total of five sampling campaigns were conducted at different stages of the reconstruction process. Every indoor sampling was supplemented with an outdoor sampling conducted near the respective building. More detailed information on the samplings is shown in Table 1.

**Figure 1:**
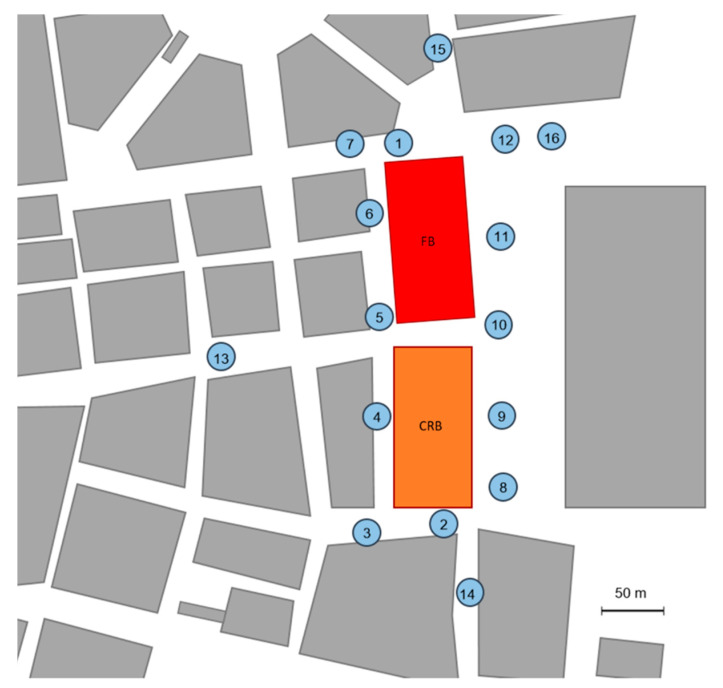
The schematic figure of the buildings and the outdoor sampling points. Sampling points (1–16) were investigated after the disinfection procedures. Yellow, CRB-labeled building is the “complete reconstruction” building, and Red, FB-labeled building is the “facadism” building.

During the reconstruction (4^th^ sampling), doors and windows were removed from the FB building. Before the 4^th^ and 5^th^ sampling campaigns, disinfection procedures were implemented throughout the entire interior of the buildings by a combined disinfectant (0.05% NaOH, 1.0% NaCl, <0.005% H_2_O_2_, <0.005% ClO_2_, SteriClean Industry, Pannon-Trade Ltd., Győr, Hungary) using dry fogging technique at 60 L/h flow rate (droplet size < 100 μm, fogging distance 80–100 m, capacity: 4000 m^3^/h) with K-30 portable thermal foggers (pulsFOG Dr. Stahl & Sohn GmbH, Überlingen, Germany). During the disinfection, plastic sheets were applied to seal the openings (removed doors and windows). Samples were collected 3 days after the disinfection procedures (8 December 2022 and 23 February 2023). To estimate the effect of the reconstruction works on the outdoor air quality, outdoor samples were also collected around the two buildings during the 4^th^ (9 December 2022 10:10–11:30) and the 5^th^ sampling (23 January 2023. 11:35–12:55) campaigns. The schematic map of the buildings and the outdoor sampling points are shown in Figure 1.

For the detection and quantification of airborne fungi, a culture-based method was used. Cultivation techniques can provide data on viable spores, so the effectiveness of disinfection can be tested. These methods are highly sensitive and enable the identification of a wide variety of species. However, quantifying viable microorganisms presents certain limitations, the most important of which is often poor reproducibility due to short sampling times and highly variable concentrations. To address the limitations of this approach, as in many exposure studies, we also incorporated surface sampling, which is generally less influenced by temporal fluctuations [17]. Air samples were collected using the IUL Spin Air microbial air sampler (IUL SA, Barcelona, Spain). This device contains a one-stage impactor plate with 400 holes, operating at a flow rate of 100 L min^−1^, and collects samples onto blood agar (5 g bacteriological peptone, 10 g casein peptone, 5 g yeast extract, 5 g NaCl, 13 g agar, 1000 mL deionized water, 50 mL sterilized sheep blood (Novagél Ltd., Karcag, Hungary) and 2% malt extract agar [18] supplemented with 0.1 g L^−1^ chloramphenicol. The sampling time was 1 min in each case. The device was sterilized with ethanol between each sample collection. The samples were incubated at 37 °C for three days for the heterotrophic counts and at 25 °C for 5–7 days for the total fungi, including all identified and non-identified CFUs. After incubation, the colony-forming units were counted, and the concentrations (CFU m^−3^) were calculated using the ‘positive hole correction’ method [19,20]. Fungal species were identified based on morphological characteristics and assessed on lactophenol cotton blue-stained colony samples. Observations included colony features such as color, shape, and hyphal dimensions, as well as structures like conidiophores and conidia. Identification was conducted using a light microscope at 600× magnification (Zeiss Jenaval, Jena, Germany), with reference to established mycological literature (e.g., [21,22]).

In addition to air sampling, samples were also collected from horizontal (settled dust) and vertical (wall) surfaces of 100 cm^2^ each, applying swab samplers containing 10 mL Neutralization Broth (Noack Hungary Ltd., Budapest, Hungary). Following the surface samplings, the swabs were transported to the laboratory in ice-packed cooling bags within an hour. A total of 1–1 mL samples were used to estimate the total colony and the total fungal counts. The same medium and conditions were used for incubation as for the air samples. After incubation, the CFU values were counted, and the concentrations (CFU/100 cm^2^) were calculated using 10-fold multiplication.

**Figure 2:**
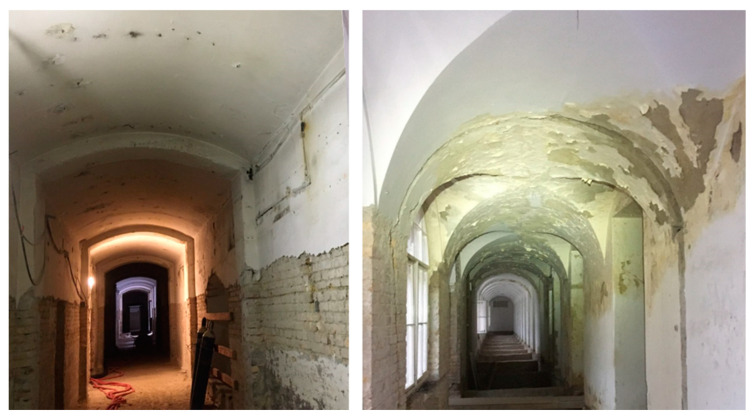
Water damage and mold colonies in the investigated buildings. (**Left**): complete reconstruction building; (**right**): facadism building.

During the sampling process, environmental parameters such as relative humidity, temperature, wind direction, and wind velocity were measured by Testo 405-V1 (Testo Hungary Ltd., Budapest, Hungary) and GFTB 200 devices (Greisinger Gmbh, Münzbach, Austria). Water-damaged walls and visible mold growth were detected in each building (Figure 2).

Further, 23 outdoor samples were collected from various distant (1–20 km) locations within Budapest during the reconstruction period. To evaluate the potential impact of the reconstruction on these samples, the atmospheric dispersion of the spores was simulated. The applied atmospheric dispersion model was developed by the authors and has been applied previously for various dispersion simulations [23,24]. The model applied the Lagrangian trajectory approach, taking into account spatially and temporally dependent wind transport and turbulent dispersion [25]. Trajectories were initiated at 10-s intervals between 0–100 m altitude above the reconstruction site. Meteorological data were obtained from the Global Data Assimilation System (GDAS) of the National Oceanic and Atmospheric Administration (NOAA) at 0.25° temporal and 3-hourly spatial resolution. As the emission rate of the spores was unknown, the dispersion simulation produced sensitivity maps in units of Q/m^3^, where Q is the (unestimated) source rate of spores. This approach enabled us to distinguish between in-plume and out-plume samples despite being unable to quantify the contribution of the reconstruction to sampled CFU values. The relationship between indoor- and outdoor-specific spores was investigated separately for in-plume and out-plume samples to identify any potential indoor-related surplus in in-plume samples, which could be attributed to the reconstruction. For this purpose, the Environmental Relative Moldiness Index (ERMI) was adopted and calculated for each sample, as defined by Täubel et al. [26].

## 3. Results

During the sampling campaign, relative humidity varied between 40% and 83% inside the buildings, while temperature values were between 5 °C and 26 °C. The environmental parameters measured during the samplings are shown in Supplementary Table S1. A total of 70–70 air samples, 60–60 surface samples, and 4–4 dust samples were collected in both historical buildings. For the 4^th^ and 5^th^ sampling campaigns, 16–16 outdoor air samples were collected for fungi, resulting in 32 samples overall. In 1–20 km locations, a total of 23 air samples were collected. During the sampling campaigns, concentrations of airborne heterotrophs and fungi were measured both outdoors and inside the CRB and FB buildings over several sessions. The primary goal was to observe how fungal levels and types changed throughout the reconstruction works, especially following disinfection treatments.

### 3.1. Results of the Complete Reconstruction Building (CRB)

#### 3.1.1. Heterotrophic Concentration in CRB

**Figure 3:**
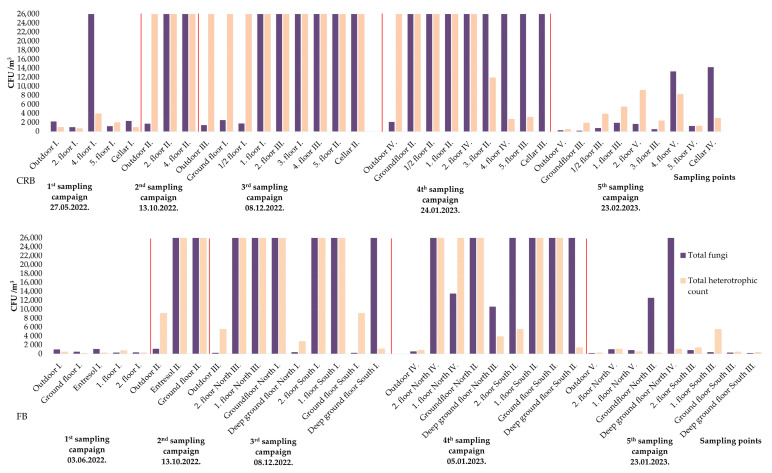
Total fungal and heterotrophic concentrations in CRB and FB during the sampling campaigns. CRB = complete reconstruction building, FB = Facadism building. Red lines separate the sampling campaings.

The heterotrophic concentration was relatively low during the 1^st^ sampling campaign, with 1000 CFU/m^3^ detected in the outdoor air and 700–4000 CFU/m^3^ in the indoor air. During the 2^nd^ and 3^rd^ sampling campaigns, every sample reached the detection limit of the method (26,280 CFU/m^3^) (Figure 3). After the first disinfection procedure (4^th^ sampling campaign), the heterotrophic concentration remained above the detection limit in the outdoor air, although 42.8% of the indoor sampling points showed lower CFU values (2770–11,890 CFU/m^3^). After the second disinfection (5^th^ sampling campaign), there was a notable reduction in outdoor concentration (480 CFU/m^3^), while the concentration also decreased at each indoor sampling point (1280–5530 CFU/m^3^).

#### 3.1.2. Total Fungal Concentration in CRB

The descriptive statistics of the fungal concentrations are shown in Table 2.

In the 1^st^ sampling campaign, the outdoor fungal concentration was 2190 CFU/m^3^, while the indoor air contained 920–2340 CFU/m^3^. One sampling point (4^th^ floor) showed an elevated value: 26,270 CFU/m^3^ (Supplementary Figure S1). In these initial samples, *Cladosporium* was the most common genus in the outdoor air and was also detected indoors, found to be dominant on the 2^nd^ and 5^th^ floors of the CRB. Various *Aspergillus* species were identified indoors at the beginning of the demolition process: *A.* sect. *Circumdati* (outdoor and cellar sampling point), *A.* sect. *Nigri* (4^th^ floor), and *A.* sect. *Fumigati* (cellar). At two sampling points (4^th^ floor and cellar), *A.* series *Versicolores* was the most common mold. Additionally, *Penicillium* showed up in high amounts on the 4^th^ floor alongside *Aspergillus* (Supplementary Figure S1).

In the 2^nd^ sampling campaign, the outdoor total fungal concentration was 1720 CFU/m^3^, while indoor concentrations generally reached the detection limit of the method (Figure 3). Outdoors, *Cladosporium* and *Penicillium* continued to be prominent. Indoors, *Penicillium* on the 2^nd^ floor and *A.* series *Versicolores* on the 4^th^ floor were the most common fungi detected (Supplementary Material Figure S2).

The characteristics of the outdoor and indoor air have changed by the 3^rd^ sampling time: *Cladosporium* levels declined, and *Penicillium* became more prominent. The outdoor total fungal concentration was 1440 CFU/m^3^, and indoor concentrations reached the detection limit at most sampling points (Figure 3). *Aspergillus* series *Versicolores* was not detected; however, *A.* sect. *Nigri* appeared in high concentrations at one sampling point (5^th^ floor) (Supplementary Figure S3).

After the first disinfection procedure, the 4^th^ sampling campaign was conducted. The outdoor air contained 2110 CFU/m^3^ fungi. Indoors, high fungal concentrations persisted, continuing to reach the detection limit at many points despite the disinfection (Figure 3). The composition of the fungal community had not changed markedly: *Penicillium* was still the most common genus indoors, while *Aspergillus* spp., especially *A.* sect. *Nigri* was present throughout the building. In the cellar, the dominant taxa belonged to another *Aspergillus* spp. (Supplementary Figure S4).

Following a second disinfection, the 5^th^ sampling campaign showed a substantial decrease in concentrations. The total fungal concentration dropped to 280 CFU/m^3^ outdoors and 160–14,200 CFU/m^3^ indoors (Figure 3). Following the second disinfection, the composition of the taxa also changed: *Cladosporium* spp. was still dominant in the outdoor air, but *Penicillium* spp. and *A*. *sydowii* were also present in elevated values. Inside the building, *Penicillium* spp. were dominant, but different *Aspergillus* spp. were also detected: *A*. *sydowii*, *A*. sect. *Fumigati*, *A*. sect. *Nigri*, and *A*. sect. *Nidulantes* (Supplementary Figure S5).

In addition to the air samples, surface and dust samples were also collected. The fungi detected in the air samples were present in the surface and dust samples, i.e., when *Penicillium* spp. showed the highest CFU values in the air samples; these fungi were also present in the surface and dust samples, indicating a persistent source of air pollution (Table 3).

### 3.2. Results of the Facadism Building (FB)

#### 3.2.1. Heterotrophic Concentration in FB

The heterotrophic concentration during the 1^st^ sampling campaign was 480 CFU/m^3^ in the outdoor air and 180–770 CFU/m^3^ in the indoor air. However, the outdoor air samples taken during the 2^nd^ and 3^rd^ sampling campaigns showed 9170 and 5530 CFU/m^3^ heterotrophic concentrations. Inside the building, most CFU values reached the detection limit of the method (Figure 3). After the first disinfection procedure (4^th^ sampling campaign), outdoor air contained 890 CFU/m^3^. Inside the building, the heterotrophic concentration decreased at 43% of the sampling points (1420–5530 CFU/m^3^). After the second disinfection (5^th^ sampling campaign), heterotrophic concentrations decreased again at every sampling point: CFU values were 310 CFU/m^3^ in the outdoor air and 290–5530 CFU/m^3^ in the indoor air samples.

#### 3.2.2. Total Fungal Concentration in FB

The descriptive statistics of the fungal concentrations are shown in Table 2.

In the 1^st^ sampling campaign, the total fungal concentration was 970 CFU/m^3^ outdoors and 300–1090 CFU/m^3^ indoors (Figure 3). The most common genera were *Cladosporium* and *Penicillium* in the outdoor air sample, while only low concentrations of *Aspergillus* spp. (*A*. sec. *Nigri*, *A*. series *Versicolores*, and *A*. sect. *Fumigati*) were found (Supplementary Figure S6).

By the 2^nd^ sampling, the composition of the air samples had changed, and indoor concentrations had substantially increased. *Penicillium* and *Aspergillus* spp. became the dominant mold, particularly in the indoor air, while *Cladosporium* spp. levels decreased. The total fungal concentration was 1150 CFU/m^3^ outdoors, but all indoor samples reached the detection limit of the method (Figure 3). The outdoor sample showed high *Cladosporium* but also elevated *Aspergillus* spp. levels. Inside the building, *Aspergillus* and *Penicillium* spp. were the dominant molds (Supplementary Material Figure S7).

In the 3^rd^ sampling, the outdoor total fungal concentration was only 270 CFU/m^3^, dominated by *Cladosporium* and *Penicillium* spp., but *Aspergillus* spp., including *A. sydowii*, was also detected. Samples taken inside the building reached the detection limit at almost every indoor sampling point (Figure 3). Indoors, the most common fungi were *Penicillium* spp., but different *Aspergillus* species were also present (*A*. *sydowii*, *A*. sect. *Nigri*, and *A*. sect. *Fumigati*) (Supplementary Figure S8).

Following the first disinfection (4^th^ sampling campaign), the outdoor total fungal concentration was 520 CFU/m^3^, but indoor levels remained high, over 26,280 CFU/m^3^ in most areas (Figure 3). The composition of the fungal community remained largely unchanged, with *Penicillium* showing the highest concentrations and *Aspergillus* (*A.* sect. *Nigri* and *A.* sect. *Fumigati*) also present indoors (Supplementary Figure S9).

After the second disinfection (5^th^ sampling campaign), however, outdoor and indoor total fungal concentrations decreased substantially, to 200 CFU/m^3^ outdoors and 190–1010 CFU/m^3^ indoors (Figure 3). Meanwhile, the second disinfection had no substantial impact on the taxa composition, with *Penicillium* spp. continuing to show the highest CFU values and only *A*. *sydowii* present in the genus *Aspergillus* (Supplementary Figure S10).

Surface and dust samplings showed that fungi detected in the air samples were also present in the surface and dust samples, indicating the persistent source of air pollution. *Penicillium* spp. showed the highest CFU values, but *Aspergillus* species were also present (Table 4).

### 3.3. Results of the Outdoor Air Sampling Points Around the Buildings After Disinfection Procedures

The descriptive statistics of the fungal concentrations are shown in Table 2.

In the case of CRB, predominantly *Cladosporium* spp. were detected (91% of total fungi) at the beginning of the samplings (27 May 2022). The taxa composition has changed as the reconstruction works progressed, in addition to *Cladosporium* spp. (71%) *Penicillium* (28%) species appeared (13 October 2022). By the 3^rd^ sampling time (8 December 2022), *Penicillium* spp. showed higher CFU values (32% of the total fungi).

*Cladosporium* spp. were also the dominant species (55% of total fungi) during the 1^st^ sampling time (3 June 2022) of the FB building, although *Penicillium* spp. was also present (12%). The ratio of the fungal species has changed during the renovation process; in addition to *Cladosporium* spp. (48%), the occurrence of *Aspergillus* spp. (34%) also became common (13 October 2022). By the 3^rd^ sampling time (8 December 2022), CFU values of *Aspergillus* spp. substantially decreased, while *Cladosporium* spp. (44%), and *Penicillium* spp. became dominant (33%).

After the first disinfection procedure, several outdoor samples were collected (9 December 2022) to investigate the effect of the reconstruction works on the outdoor air quality around the buildings. For this purpose, a total of 16 sampling points were selected at different distances from the buildings (Figure 1). Table 4 shows the total colony and fungal counts and the most common fungi of the 16 sampling points after the disinfection procedures (Table 5).

After the first disinfection, *Penicillium* spp. was detected inside the buildings and also showed high CFU values in the outdoor air, particularly in samples collected in front of the buildings (1G, 8–12G, 14G) and at one additional sampling point (13G). *Penicillium* spp. were present in every outdoor sample. With regard to the *Aspergillus* species, our outdoor air samples contained species of potentially indoor origin, i.e., *A*. sect. *Nigri* and *A*. *sydowii* (Supplementary Figure S11).

While there was little change in the total CFU values during the disinfection procedures (especially after the first disinfection), the composition of the outdoor air samples differed markedly after the second disinfection. *Cladosporium* spp. were dominant again at almost every sampling point. Only one sampling point showed high *Penicillium* CFU values (11G). Interestingly, *Geotrichum* spp. at 13G and *Paecilomyces* spp. at 16G sampling points were dominant. *Penicillium* spp. and *Aspergillus* species (*A*. sect. *Nigri*, *A*. *sydowii*, and *A*. sect. *Fumigati*) also appeared with low CFU values in the outdoor sampling points (Supplementary Figure S12) when these fungi had high indoor concentrations.

### 3.4. Possible Urban Scale Dispersion of the Released Fungi

**Figure 4:**
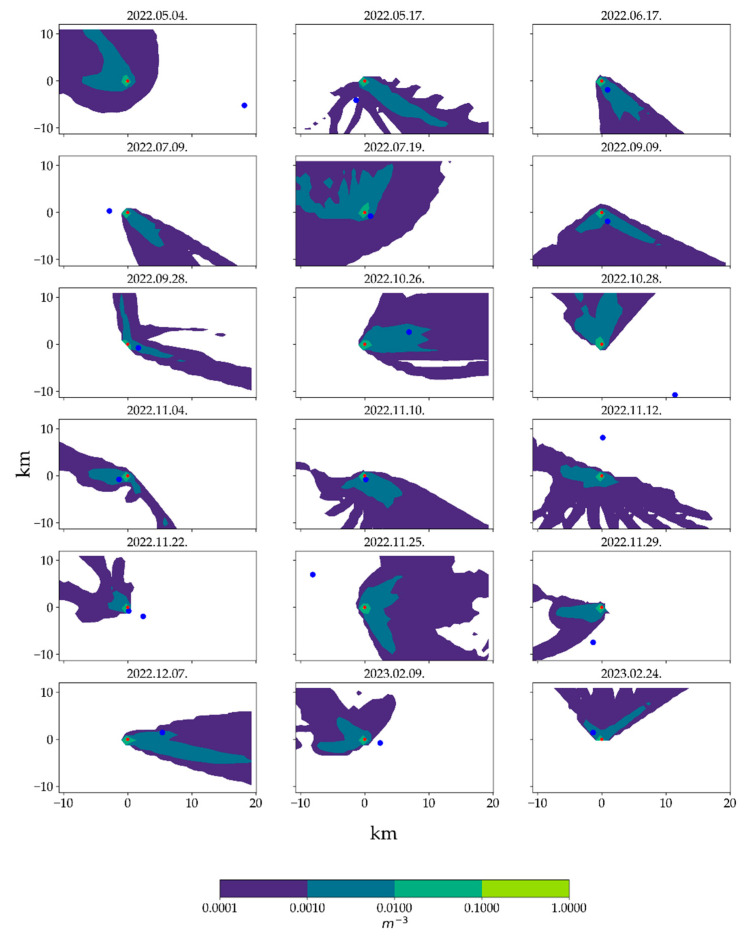
Daily atmospheric dispersion plumes, calculated by a Lagrangian model. Red dots: construction area; blue dots: air sampling points.

During the reconstruction period, a total of 23 outdoor air samples were collected from various locations within Budapest, situated at a 1–20 km distance from the reconstruction site (Table 6). An atmospheric dispersion model was applied to assess the potential wind transport of spores from the reconstruction site to the sampling point on a specific day. Based on dispersion model results, samples were separated into two groups: 12 samples that were unaffected by the reconstruction plume (out-plume) and 8 samples that were possibly affected by the reconstruction (in-plume). Three samples were not considered due to the occurrence of precipitation (>5 mm) on the given day. Maps showing the daily dispersal plumes are shown in Figure 4.

**Figure 5:**
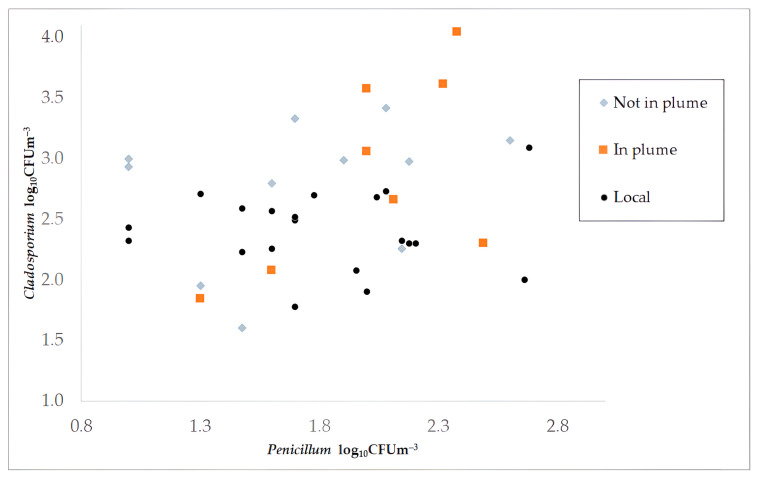
*Penicillum* and *Cladosporium* logarithmic concentrations in outdoor air samples taken in Budapest during the reconstruction period. Grey marks show samples taken at >1 km distance and unaffected by the plume of the reconstruction, according to the results of the atmospheric dispersion simulation. Orange marks show samples located at >1 km distance but within the dispersion plume. Black marks indicate local samples taken within a 1 km distance at locations indicated in Figure 1.

Due to the impact of multiple other emissions on these distant samples and the inability to quantify the spore release rate of the reconstruction, our focus shifted to examining the relationship between spores specific to the outdoor environment (represented by *Cladosporium* spp.) and those specific to the indoor environment (represented by *Penicillum* spp.). This approach aimed to assess the potential building-originated contribution to the urban background outdoor concentrations. To support this analysis, the ERMI index was calculated for each sample. Results are presented in Figure 5, indicating in-plume and out-plume samples. For comparison, local samples, presented in Section 3.3, are also shown in the figure. Although some rightward (i.e., more *Penicillum*) shift is perceptible in the in-plume (orange) group compared to the out-plume (grey) points in Figure 5, the separation is weak.

Statistical tests also failed to detect significant separation between in-plume and out-plume samples. The mean and the standard deviation of *Cladosporium* concentration were 991 ± 820 CFU/m^3^ out-plume and 2620 ± 3791 CFU/m^3^ in-plume. In the case of *Penicillum*, 95 ± 113 CFU/m^3^ was obtained out-plume and 144 ± 101 CFU/m^3^ in-plume. The ERMI index was −36 ± 28 out-plume and −31 ± 28 in-plume. The large variability of concentrations inhibits the attribution of the small separation in the means. The Kolmogorov–Smirnov statistics were 0.38 (*p*-value 0.45) for *Cladosporium*, 0.42 (*p*-value 0.32) for *Penicillum*, and also 0.42 (*p*-value 0.32) for ERMI. ANOVA test yielded an F-statistic of 2.6 (*p*-value 0.12) for *Cladosporium*, 0.18 (*p*-value 0.68) for *Penicillum*, and 0.37 (*p*-value 0.55) for ERMI. The MANOVA test for the two-dimensional separation between *Cladosporium* and *Penicillum* resulted in an F-statistic of 0.88 (*p*-value 0.43). Insignificant results (*p* > 0.05 in all cases) show that the impact of the reconstruction remained within the range of urban background variability at distant (>1 km) locations, although it caused local microbial pollution, often exceeding the detection limit in near-site samples.

## 4. Discussion

This study investigated two historical buildings that were approximately 100 years old. During the initial phase of the renovation procedures, we collected air, surface, and dust samples. It was demonstrated that extremely high concentrations of fungi can be measured during reconstruction works in the indoor air. This is possibly due to the release of fungal spores in tremendous numbers by the handling of old, polluted, or moldy building materials. In addition to the indoor air, the outdoor air was also found to be significantly contaminated with fungal aerosols, potentially due to construction waste (the removal of doors and windows from the FB may also have allowed spores to leave the building). The concentration of fungi increased rapidly throughout the course of the work. In the case of CRB, the initial values of heterotrophic concentrations were found to be 2–4 times higher in the indoor samples compared to the outdoor sample collected next to the building. During the reconstruction of CRB, the CFU values reached extremely high levels (i.e., above the detection limit of the method) inside the building. Unlike FB, the windows of the CRB remained closed. Therefore, the emissions from FB possibly did not have any major effect on the indoor air quality of CRB. Surface sampling within both buildings confirmed the presence of indoor spore sources, supporting the assumption that cross-contamination between the two buildings was minimal. Thus, the indoor air quality in both CRB and FB primarily reflects their respective internal sources. Given the closed windows in CRB, spore emissions from this building to the outdoor environment were likely lower than those from FB. 

High concentrations of airborne fungi can adversely impact human health in four primary ways: (i) acting as allergens, (ii) causing infections, (iii) triggering inflammatory reactions, and (iv) producing toxins [27]. To mitigate health risks, disinfection was conducted in the buildings to reduce high fungal concentrations. Following the second disinfection procedure, heterotrophic concentrations decreased significantly. However, indoor CFU values remained 44% higher than at the outset of the reconstruction process. In the case of facadism, initial heterotrophic concentrations were lower than those observed in the CRB case. However, during the renovation process, several sampling points reached the detection limit.

The demolition processes resulted in alterations to the total fungal counts and the composition of fungal species. Initially, total counts were lower both inside and outside, but during the renovations, total fungal counts were 2–4 times higher in the air samples. Initially, air samples indicated high CFU values for *Cladosporium* spp. However, as the demolition progressed, there was a shift in dominance. It should be noted that *Cladosporium* spp., which is typically found in outdoor air, is not pathogenic to humans. However, prolonged exposure may result in the development of an allergic reaction [3]. The indoor air samples revealed the presence of several pathogenic species, with concentrations increasing during the demolition process. *Penicillium* spp. were the most prevalent fungi in both buildings and are primarily known to cause respiratory allergies, rhinitis, and hypersensitivity pneumonitis [3,28]. Elevated concentrations of *Penicillium* spp. in outdoor air are frequently observed in proximity to construction sites where building materials and soil undergo extensive disturbance (Donát Magyar, unpublished data). However, *Penicillium* is generally more prevalent in indoor air than in outdoor air, with many common species of this genus considered indicators of moisture damage. Environmental factors, including elevated relative humidity and lower temperatures, are thought to contribute to the increased indoor presence of these fungi [26]. In both the CRB and FB buildings, the relative humidity and temperature were optimal for fungal proliferation. In addition, *Penicillium* spp. release spores more easily than other fungi, such as *Aspergillus* series *Versicolores* [28], and this is probably why Penicillia were more prevalent than Aspergilli.

As our results showed, different *Aspergillus* species could also proliferate inside the two buildings, but their ratio and species composition were different (Supplementary Table S2). *Aspergillus* can cause pathologies too that specifically affect the respiratory system. Inhalation can result in the infection of the lungs, skin, heart, brain, and kidneys, particularly in individuals with compromised immunity [3]. The composition and concentration of *Aspergillus* species were found to vary during the course of the sampling campaigns. However, the same species were consistently detected in the buildings at different times. *Aspergillus* sect. *Circumdati* includes industrially important species, while others can produce various mycotoxins [29] on ceiling tiles and carpets [30]. Members of the *A*. series *Versicolores* are typically found in the indoor environment. These species can cause allergy, asthma, and pulmonary aspergillosis, and they are involved in the sick-building syndrome, too. They can produce different mycotoxins, e.g., sterigmatocystin, a mycotoxin involved in the aflatoxin biosynthetic pathway and recognized as a potential carcinogen [31]. The fungus *Aspergillus sydowii* is known as a mesophilic soil saprobe, which is a food contaminant and an opportunistic pathogen of humans [32]. Members of *Aspergillus* sec. *Nigri* is known as black aspergilli. They are among the most extensively researched fungi, playing a pivotal role in food, medical, and biotechnological mycology. Due to their metabolic properties, these fungi are widely used in industry. However, they are also responsible for several human diseases of the respiratory tract and ear canal. *Aspergillus niger* is one of the most prevalent causative species of aspergillosis in humans, following *A. fumigatus*. It is also known that they can produce carcinogenic mycotoxins [33,34]. Members of *Aspergillus* sect. *Fumigati* are opportunistic human pathogens and allergenic molds that can cause food spoilage and produce mycotoxins [35]. This species is most commonly involved in invasive aspergillosis in immunocompromised, hospitalized patients [16]. *A*. sect. *Nidulantes* species are widely distributed in nature and play a significant role in decomposition processes, although it has been demonstrated as a human pathogen causing different infections [36]. Further studies should focus on the molecular identification of species aerosolized by demolition works, their endo- and mycotoxin production/levels, and the assessment of the health risk.

It should be highlighted that indoor species could cause high pollution of the outdoor air during the demolition procedures. Microorganisms detected inside the buildings could be detected around the buildings, too: *Penicillium* spp. showed high CFU values at multiple sampling points, but also *Aspergillus* species were present in the outdoor air. After the second disinfection, the fungal composition substantially changed, although pathogenic fungi were still detected. Two outdoor sampling points (13G and 16G) showed different fungi (*Geotrichum* and *Paecilomyces* spp.). It can be assumed that their source is not in the renovated buildings. *Geotrichum* species are common soil-borne fungi with higher prevalence in sewage-polluted soils. *Paecilomyces* spp. are frequently isolated from composts [18,37] and water-damaged wooden flooring of old buildings (unpublished observation of Donát Magyar). We suppose that these fungi were aerosolized due to the disturbance of the soil surrounding the buildings.

Emissions from construction sites, in the absence of wind erosion control and dust suppression measures, can be several orders of magnitude higher than background levels observed prior to these activities [38]. Pollution from demolition activities can have adverse effects on the health of individuals living near demolition sites, particularly when measures to control the release of particles are insufficient. Demolition sites are often located in highly urbanized areas where it is challenging to fully comply with regulatory requirements or adhere strictly to relevant guidelines. Prevention of air pollution becomes even more critical when these sites are situated in densely populated residential areas or in proximity to sensitive locations such as schools and hospitals [38]. In line with our results, previous studies have reported associations between fungal contamination and building demolition with increased spore emissions. As mainly hospital studies are available, the risk of fungal infections in hospitalized (immunocompromised) patients due to hospital demolition or construction is emphasized in the literature. Loeffert et al. detected *Aspergillus fumigatus* indoors and around a hospital, and they concluded that clinical infection and colonization may originate from hospital environments during the demolition works [16]. The release of *Aspergillus* species into the air due to implosion and demolition of hospitals was demonstrated [11]. Other studies [12,13,14] showed high CFU values of *Aspergillus* spp. during hospital construction and renovation. Sautour et al. reported increasing concentrations of airborne *Penicillium* and *Aspergillus* spp. during a hospital demolition [15]. All hospital studies have highlighted the risks associated with renovation: outbreaks of invasive aspergillosis among hospitalized patients have been associated with demolition and building construction [11,12,13,14,15,16]. Kanamori et al. analyzed data about fungal outbreaks that were related to hospital demolition over the last four decades. They identified different *Aspergillus* species that caused hospital infections due to the spores’ elimination during demolition works. They proposed a series of control measures to prevent fungal contamination, including maintaining hospital ventilation system, removing patients from renovating areas, and applying physical barriers and face masks [39].

Although *A.* sect. *Fumigati* (probably *A*. *fumigatus*) was detected in our study during the renovation of historical buildings, they were not the dominant species (their max. concentration was 20 CFU/m^3^). However, considering the pathogenicity of this fungus, their increased prevalence is obviously a risk factor for non-hospitalized but sensitive populations living in the proximity of facadism or complete reconstruction buildings. Some interventions can be adapted from hospital studies [13,14,16], e.g., instructing workers on dust control methods, spraying water on the work area to wet the dust particles (making them heavier and less likely to become airborne), sealing windows of nearby buildings and avoiding their natural ventilation during the main demolition (high dust) periods, and informing the potentially exposed local population to wear FFP3 (N95)-grade safety masks if susceptible to organic dust and spores (immunocompromised persons, asthmatics, people with chronic obstructive pulmonary disease/COPD). It should be noted, however, that 20 CFU/m^3^ concentration of *A. fumigatus* in outdoor samples in Hungary is not rare, and their origin from composting piles (their typical habitat) is more likely than from construction works (unpublished data of the authors).

Although the pathogenic *Aspergillus fumigatus* is of great importance, other fungi, like *A.* sect. *Nigri*, *A*. series *Versicolores*, and *Penicillium* spp., can also pose a health hazard, especially due to their allergenic properties and high concentration. Such fungi were released during the reconstruction of historic buildings in high quantity. Our findings indicate that the demolition and renovation process can disturb the loci where fungal spores are accumulated in old buildings. During renovation, common airborne molds are replaced by typically indoor species, which can reach high concentrations in and near the building. As demolition progresses, the composition of emitted fungal aerosol changes, possibly due to the disturbance of different building materials (plaster, wallpaper, wood, soil, etc.) that harbor different types of fungi (see figures in the Supplementary Materials). Our findings on species composition are comparable with the data presented by Hameed et al. [40] in non-hospital buildings (laboratories, offices) undergoing renovation in Egypt. The authors analyzed the concentration of fungi in the suspended dust and air samples using gravitational sedimentation, both before and during the renovation process. During the renovation period, *Aspergillus* spp., *Penicillium* spp., *Eurotium* spp., and *Paecilomyces* spores were identified in suspended dust samples, while air samples contained *Aspergillus*, *Cladosporium*, *Alternaria*, and *Penicillium* species. This species composition differs somewhat from our results, possibly because of the warmer climate and the selective effect of the gravitational sedimentation sampling method on larger spores [41].

Hyvönen et al. studied the relationship between fungal exposure and the workers’ asthma in a water-damaged building during an 8-month renovation work of an office building in Finland. They showed that 7.2% of workers developed new-onset asthma, and after one year, 61.9% of patients continued to experience symptoms. Their results showed that several mold species and an increased presence of pathogenic fungi could be detected during the renovation of non-hospital buildings [41]. To avoid health problems, the proposed intervention can be spraying water on the work area and instructing workers to wear N95-grade safety masks during the renovation [14].

Biocides, mechanical treatments and physical treatments are existing techniques in heritage buildings to control microbial contamination. Abdel et al. studied the effectiveness of biocides on fungal taxa in surface and air samples too in a historical museum. Samples were collected before and after the treatment. The dominant fungal species were *Aspergillus niger*, *A. fumigatus*, and *A. flavus*; however, high-water activity fungi were also detected. After various biocide treatments, fungal counts increased, and the diversity of samples also altered due to the different killing mechanisms and targets of the biocides [42]. During our studies, disinfection was applied twice in both buildings by fogging. After the first disinfection, concentrations did not decrease, neither in FB nor in CRB. Heterotrophic concentrations decreased substantially only after the second disinfection in both buildings. The failure of the first disinfection can likely be attributed to the presence of a thick layer of dust settled on the horizontal surfaces. This layer was composed of particles from demolished building materials (e.g., debris from plaster, wall paint, and flooring) and spores of fungi that had colonized the walls before the reconstruction. The droplets of the disinfectants probably wetted only the surface of the thick dust layer, harming no spores that were deeper in the dust. These spores were still viable and stirred up with the dust when the reconstruction works continued after the disinfection episode, producing high bioaerosol concentrations again. For a more effective reduction of bioaerosol levels, one should consider: i. disinfect moldy surfaces and remove wall paint and plaster while still wet to avoid spore aerosolization and spread; ii. remove a thick layer of settled dust before fogging. It should be noted that a wide variety of contaminated building materials can be present in buildings, on which either visible or hidden mold can develop. Consequently, effective decontamination during the demolition process is challenging, and achieving zero emissions of bioaerosols is difficult, if not impossible. Further research is required to develop effective methods to reduce emissions of (bio)aerosols during building demolition. Disinfection may be more effective in later phases of reconstruction once new, cleanable surfaces, such as ceramic flooring, have been installed [38].

Besides indoor air quality, we performed samplings to study the impact of reconstruction works on the quality of outdoor air. In atmospheric studies on particulate matter (PM), there is substantial evidence that activities such as building renovations, earthmoving, and demolition degrade the surrounding air quality. In near-source environments, dilution of atmospheric pollutants imposes sharp spatial gradients for directly emitted air pollutants [43], and downwash turbulence creates complex concentration patterns. Therefore, single near-source samples are barely representative of the local conditions. We defined 16 near-source sampling points to capture the spatial variability of the local pollution and found variability among measured concentrations reaching a factor of 10. However, even more sampling points and/or sophisticated street-scale flow simulations would be required to continuously explore the complex spatial patterns in a densely built historical environment, also impacted by other local sources.

On the urban scale of 1–20 km, the concentrations downwind from the source typically follow a Gaussian distribution, although this is altered by the complexities of the urban landscape [25,44]. On this scale, surface concentrations are largely impacted by the mixing layer height, i.e., the depth of the layer in which pollutants are mixed by turbulence either created thermally (by air uprising from the warm surface) or mechanically (by eddies formed around rough surface obstacles) [45]. Due to differences in surface temperature and hence the convective mixing, the mixing layer is substantially deeper in the summer (1000–2000 m) than in the winter (100–500 m), which causes larger near-surface concentrations of pollutants in the winter due to limited vertical mixing. Although we performed samplings from May 2022 to February 2023, no seasonality was found in the results, further indicating that the observed concentrations are due to local sources and are largely independent of urban-scale atmospheric dispersion processes.

A main advantage of the current study is the high level of comparability provided by the same climatic environment, almost the same period, and the similar size and age of the buildings. On the other hand, our study has some limitations. The culture-based method used in this study has a limitation in that it selectively detects certain species and does not identify non-culturable or dead microorganisms or their fragments, although they, too, may have toxic or allergenic properties [17]. To test the effectiveness of disinfection, a culture-based approach was applied. Combining this approach with microscopy and/or DNA-based sampling methods could provide a more comprehensive understanding of species composition. Although for the detection of total heterotrophs, TSA or TSA-lecithin agar is more commonly used, blood agar, applied in this study, has the potential to detect airborne pathogens [46]. Although a standard sampling method (100 L) was applied, our samplings resulted in concentrations above the technological detection limits due to the high levels of biological air pollutants. Nakanishi et al. [14] reported “too many colonies to count” for most of their outdoor samples collected during demolition work in Japan. Other data from similar study settings on the airborne concentration of fungi are not available in the literature (in hospital demolition studies, windows were sealed, and indoor samplings resulted in lower levels of fungi, while outdoor concentrations were not reported). In most studies similar to the present one, a cultivation-based sampling method was used, which can reach a detection limit. In further research, serial dilution of air samples collected by an impinger should be used, which may provide more precise quantitative data. Although we were not able to determine the exact CFU levels, it does not change the essence of the message that the concentrations during the reconstruction works of the historical buildings were extremely high. It is obvious that both reconstruction methods emit enormous amounts of bioaerosols, and in this aspect, the difference between the CRB and FB approaches (at least during the main work period) is negligible.

## 5. Conclusions

During renovation works of historical buildings, there is a potential for the emission of significant quantities of fungal bioaerosols into the air. Our findings indicate that there is no discernible difference between facadism and complete reconstruction of the buildings in terms of the indoor concentrations of these bioaerosols. Several pathogen species were identified in large concentrations inside the buildings, and their presence was also examined around the buildings. Although demolition procedures negatively affected the local (<1 km) outdoor air quality, the statistical analysis of the atmospheric dispersion model demonstrated that there was no significant impact on a larger geographical scale (>1 km) in the urban environment. In summary, both types of demolition procedures of historical buildings were found to have a substantial negative effect on indoor air quality, while facadism might have a major impact on near-site outdoor air quality, too.

## Figures and Tables

**Table 1:** Sampling campaigns in the complete reconstruction building and the facadism building.

Complete Reconstruction Building		Facadism Building	
Sampling	Sampling Location	Sampling	Sampling Points
1^st^ sampling 27 May 2022. 11:00–11:55	Cellar; 2^nd^ floor; 4^th^ floor; 5th floor, Outdoors 5 air samples, 4 surface samples	1^st^ sampling 3 June 2022. 10:40–11:50	Entresol, Ground floor, 1^st^ floor, 2^nd^ floor, Outdoors 5 air samples, 4 surface samples
2^nd^ sampling 13 October 2022. 13:30–14:00	2^nd^ floor; 4^th^ floor, Outdoors 3 air samples, 2 surface samples	2^nd^ sampling 13 October 2022. 14:15–14:40	Entresol, 1^st^ floor, Outdoors 3 air samples, 2 surface samples
3^rd^ sampling 8 December 2022. 14:20–16:30	Cellar; Ground floor; 1/2^th^ floor; 1^st^ floor; 2^nd^ floor; 3^rd^ floor; 4^th^ floor; 5th floor, Outdoors 9 air samples, 8 surface samples, each sampling date	3^rd^ sampling 8 December 2022. 11:20–13:30	Deep ground floor North, Ground floor North, 1^st^ floor North, 2^nd^ floor North, Deep ground floor South, Ground floor South, 1^st^ floor South, 2^nd^ floor South, Outdoors 9 air samples, 8 surface samples, each sampling date
4^th^ sampling 24 January 2023. 10:25–11:10		4^th^ sampling 5 January 2023. 10:10–11:45	
5^th^ sampling 23 February 2023. 13:10–14:40		5^th^ sampling 23 January 2023. 10:15–11:20	

**Table 2:** The descriptive statistics (median, mean, standard deviation, and maximum) of the airborne fungi.

	CRB				Outdoor CRB				FB				Outdoor FB				Outdoor Directly to the Buildings 1st Sampling				Outdoor Directly to the Buildings 2nd Sampling			
	Median	Mean	SD	Max.	Median	Mean	SD	Max.	Median	Mean	SD	Max.	Median	Mean	SD	Max.	Median	Mean	SD	Max.	Median	Mean	SD	Max.
*Acremonium* sp.	0	0.00	0.00	0	0	2.00	5.16	10	0	0.00	0.00	0	0	0.00	0.00	0	0	0.00	0.00	0	0	0.00	0.00	0
*Alternaria* spp.	0	0.00	0.00	0	0	0.00	0.00	0	0	1.29	4.28	20	0	4.00	8.94	20	0	0.00	0.00	0	0	0.63	2.50	10
*Arthrinium* sp.	0	0.00	0.00	0	0	0.00	0.00	0	0	0.00	0.00	0	0	0.00	0.00	0	0	0.00	0.00	0	0	1.25	3.42	10
*Aspergillus* sect. *Circumdati*	0	1.33	7.35	40	0	2.00	4.08	10	0	0.00	0.00	0	0	0.00	0.00	0	0	0.00	0.00	0	0	0.00	0.00	0
*Aspergillus* sect. *Flavi*	0	0.00	0.00	0	0	0.00	0.00	0	0	0.00	0.00	0	0	0.00	0.00	0	0	0.63	2.50	10	0	0.00	0.00	0
*Aspergillus* sect. *Fumigati*	0	0.67	2.50	10	0	0.00	0.00	0	0	0.65	3.59	20	0	6.00	8.94	20	0	0.00	0.00	0	0	0.63	2.50	10
*Aspergillus* sect. *Nidulantes*	0	1.00	5.39	30	0	0.00	0.00	0	0	0.00	0.00	0	0	0.00	0.00	0	0	0.00	0.00	0	0	0.00	0.00	0
*Aspergillus* sect. *Nigri*	0	1379.67	5259.37	>26,280	0	0.00	0.00	0	0	96.77	215.95	820	0	0.00	0.00	0	0	5.63	19.99	80	0	1.25	3.42	10
*Aspergillus* series *Versicolores*	0	521.03	2538.27	13,900	0	0.00	3.78	0	0	1.94	6.54	30	0	2.00	4.47	10	0	0.00	0.00	0	0	0.00	0.00	0
*Aspergillus* sp.	0	0.00	0.00	0	0	2.00	5.16	10	0	1697.74	6562.34	>26,280	0	80.00	173.35	390	0	1.25	3.42	10	0	0.00	0.00	0
*Aspergillus sydowii*	0	8.00	24.18	130	0	6.00	15.49	30	0	3.23	11.37	60	0	2.00	4.47	10	0	5.63	10.31	30	0	4.38	10.94	40
*Botrytris* spp.	0	0.00	0.00	0	0	0.00	0.00	0	0	0.00	0.00	0	0	0.00	0.00	0	0	1.25	3.42	10	0	1.88	5.44	20
*Chaetomium* spp.	0	6.67	30.83	170	0	0.00	0.00	0	0	0.32	1.80	10	0	0.00	0.00	0	0	0.00	0.00	0	0	0.00	0.00	0
*Chrysonilia sitophila*	0	3.00	4.61	10	0	2.00	22.36	10	0	0.00	0.00	0	0	0.00	0.00	0	0	0.63	2.50	10	0	1.25	3.42	10
*Cladosporium* spp.	130	348.00	576.77	2000	100	690.00	788.00	2000	130	316.13	533.00	2460	210	302.00	231.34	560	140	215.63	324.80	1420	315	355.00	272.81	1240
*Engyodontium* spp.	0	0.33	1.80	10	0	0.00	3.78	0	0	0.00	0.00	0	0	0.00	0.00	0	0	0.00	0.00	0	0	0.00	0.00	0
*Engyodontium* spp.	0	0.00	0.00	0	0	0.00	0.00	0	0	0.00	0.00	0	0	0.00	0.00	0	0	0.63	2.50	10	0	0.00	0.00	0
*Eurotium* spp.	0	0.00	0.00	0	0	2.00	5.16	10	0	0.00	0.00	0	0	0.00	0.00	0	0	0.00	0.00	0	0	0.00	0.00	0
*Fusarium* spp.	0	0.00	0.00	0	0	0.00	0.00	0	0	0.97	3.01	10	0	0.00	0.00	0	0	0.00	0.00	0	0	0.00	0.00	0
*Geotrichum* spp.	0	0.00	0.00	0	0	0.00	0.00	0	0	0.00	0.00	0	0	0.00	0.00	0	0	0.00	0.00	0	0	1645.00	6569.34	>26,280
*Mucor plumbeus*	0	0.00	0.00	0	0	0.00	0.00	0	0	0.97	5.39	30	0	0.00	0.00	0	0	0.00	0.00	0	0	0.00	0.00	0
*Mucor* spp.	0	0.67	2.50	10	0	2.00	4.08	10	0	0.00	0.00	0	0	0.00	0.00	0	0	0.63	2.50	10	0	0.00	0.00	0
*Mycotypha* sp.	0	0.00	0.00	0	0	0.00	0.00	0	0	0.65	2.50	10	0	0.00	0.00	0	0	0.00	0.00	0	0	0.00	0.00	0
*Paecilomyces* spp.	0	0.33	1.80	10	0	0.00	0.00	0	0	0.00	0.00	0	0	0.00	0.00	0	0	0.00	0.00	0	0	20.00	77.37	310
*Penicillium digitatum*	0	0.00	0.00	0	0	0.00	0.00	0	0	0.00	0.00	0	0	0.00	0.00	0	0	0.00	0.00	0	0	0.63	2.50	10
*Penicillium* spp.	13,980	14,638	12,286	>26,280	460	604.00	774.22	2030	13,240	13,911	12,430.5	>26,280	100	90.00	53.85	140	110	239.38	292.27	1050	40	1689.38	6557.68	>26,280
*Rhizopus* spp.	0	1.33	3.41	10	0	2.00	4.08	10	0	2.26	4.25	10	0	0.00	0.00	0	0	0.63	2.50	10	0	0.00	0.00	0
*Rhodotorula* spp.	0	5.33	16.30	60	0	6.00	8.16	20	0	6.45	25.37	130	0	0.00	0.00	0	15	23.13	26.26	80	0	0.63	2.50	10
*Sporothrix* sp.	0	0.00	0.00	0	0	0.00	0.00	0	0	0.00	0.00	0	0	2.00	4.47	10	0	0.00	0.00	0	0	0.00	0.00	0
*Talaromyces*	0	0.00	0.00	0	0	0.00	0.00	0	0	0.00	0.00	0	0	0.00	0.00	0	0	1.25	3.42	10	0	0.00	0.00	0
*Trichoderma* sp.	0	0.33	2.50	10	0	4.00	5.16	10	0	0.65	2.50	10	0	0.00	0.00	0	0	0.00	0.00	0	0	0.00	0.00	0
*Ulocladium* sp.	0	0.00	0.00	0	0	0.00	0.00	0	0	0.32	1.80	10	0	0.00	0.00	0	0	0.00	0.00	0	0	0.00	0.00	0
*Zygomycetes* spp.	0	1.00	3.96	20	0	0.00	0.00	0	0	0.00	0.00	0	0	0.00	0.00	0	0	0.00	0.00	0	0	0.00	0.00	0
*black yeast* spp.	0	2.00	10.78	60	0	8.00	16.32	40	0	0.97	3.01	10	0	0.00	0.00	0	0	0.00	0.00	0	0	1.25	3.42	10
*yeast* spp.	0	188.00	939.31	5240	0	166.00	311.33	780	0	30.00	77.63	350	10	16.00	20.74	50	10	20.63	32.96	130	0	6.25	9.57	30
other *coelomycetes* sp.1.	0	0.00	0.00	0	0	0.00	0.00	0	0	0.00	0.00	0	0	0.00	0.00	0	0	0.63	2.50	10	0	0.00	0.00	0
other *hyphomycetes* spp.	0	0.00	0.00	0	0	0.00	0.00	0	0	0.97	5.39	30	0	0.00	0.00	0	0	1.25	5.00	20	0	0.00	0.00	0
non *sporulating* sp.1.	0	0.00	7.18	40	0	10.00	16.02	40	0	0.00	0.00	0	0	0.00	0.00	0	0	0.00	0.00	0	0	0.00	0.00	0
non *sporulating* spp.	0	41.00	81.19	380	20	40.00	48.75	130	10	32.26	42.40	160	140	118.00	93.38	210	70	104.38	101.98	400	105	100.63	70.57	310

**Table 3:** The concentration of total fungi and the maximal concentration of the dominant taxa in the air, surface, and dust samples in the complete reconstruction building (CRB). n. s. = no sample.

Sampling Time	Outdoor Air Samples	Indoor Air Samples	Indoor Vertical Surface Samples	Indoor Dust Samples
1^st^ sampling	Total 2190 CFU/m^3^ *Cladosporium* spp. 2000 CFU/m^3^	*Cladosporium* spp. 1910 CFU/m^3^ *Penicillium* spp. 10,430 CFU/m^3^ *A.* series *Versicolores* 13,900 CFU/m^3^	*A.* series *Versicolores* 5000 CFU/100 cm^2^ *A.* sect. *Circumdati* 430 CFU/100 cm^2^	n. s.
2^nd^ sampling	Total 1720 CFU/m^3^ *Cladosporium* spp. 1230 CFU/m^3^ *Penicillium* spp. 480 CFU/m^3^	*Penicillium* spp. >26,280 CFU/m^3^ *A.* series *Versicolores* >26,280 CFU/m^3^	*Penicillium* spp. 3000 CFU/100 cm^2^ *A.* series *Versicolores* 4000 CFU/100 cm^2^	n. s.
3^rd^ sampling	Total 1440 CFU/m^3^ *Penicillium* spp. 460 CFU/m^3^	*Penicillium* spp. >26,280 CFU/m^3^	*Penicillium* spp. 10,120 CFU/100 cm^2^	n. s.
4^th^ sampling (1^st^ disinfection)	Total 2110 CFU/m^3^ *Penicillium* spp. 2030 CFU/m^3^	*Penicillium* spp. >26,280 CFU/m^3^ *A*. sect. *Nigri* 1310 CFU/m^3^	*Penicillium* spp. 3000 CFU/100 cm^2^	*Penicillium* spp. 905 CFU/100 cm^2^
5^th^ sampling (2^nd^ disinfection)	Total 280 CFU/m^3^ *Cladosporium* spp. (60 CFU/m^3^) *Penicillium* spp. 50 CFU/m^3^ *A*. *sydowii* 30 CFU/m^3^	*Penicillium* spp. 13,980 CFU/m^3^	*Penicillium* spp. 1400 CFU/100 cm^2^	*Penicillium* spp. 420 CFU/100 cm^2^

**Table 4:** The concentration of total fungi and the maximal concentration of the dominant taxa in the air, surface, and dust samples in facadism building (FB). n. s. = no sample.

Sampling Time	Outdoor Air Samples	Indoor Air Samples	Indoor Vertical Surface Samples	Indoor Dust Samples
1^st^ sampling	Total 970 CFU/m^3^ *Cladosporium* spp. 540 CFU/m^3^	*Cladosporium* spp. 290 CFU/m^3^ *Penicillium* spp. 640 CFU/m^3^	*Cladosporium* spp. 40 CFU/m^3^	n. s.
2^nd^ sampling	Total 1150 CFU/m^3^ *Cladosporium* spp. 560 CFU/m^3^ *Aspergillus* spp. 390 CFU/m^3^	*Penicillium* spp. >26,280 CFU/m^3^ *Aspergillus* spp. >26,280 CFU/m^3^	*Penicillium* spp. 12,000 CFU/100 cm^2^	n. s.
3^rd^ sampling	Total 270 CFU/m^3^ *Cladosporium* spp. (120 CFU/m^3^) *Penicillium* spp. (90 CFU/m^3^)	*Penicillium* spp. >26,280 CFU/m^3^	*Penicillium* spp. 415 CFU/100 cm^2^ *A. sydowii* 505 CFU/100 cm^2^	n. s.
4^th^ sampling (1^st^ disinfection)	Total 520 CFU/m^3^ *Penicillium* spp. 210 CFU/m^3^	*Penicillium* spp. >26,280 CFU/m^3^	*Penicillium* spp. 445 CFU/100 cm^2^	*Penicillium* spp. 165 CFU/100 cm^2^
5^th^ sampling (2^nd^ disinfection)	Total 200 CFU/m^3^ *Penicillium* spp. (100 CFU/m^3^)	*Penicillium* spp. >26,280 CFU/m^3^	*Penicillium* spp. 1300 CFU/100 cm^2^	*Penicillium* spp. 550 CFU/100 cm^2^

**Table 5:** Heterotrophic colony and fungal counts and the most prevalent fungi in the outdoor air samples after the disinfection procedures. CFU = colony-forming unit.

Sampling Point	Samplings After the First Disinfection (9 December 2022)			Samplings After the Second Disinfection (23 January 2023.)		
	Heterotrophic Concentration (CFU/m^3^)	Total fungal Concentration (CFU/m^3^)	Dominant Fungal Genera	Heterotrophic Concentration (CFU/m^3^)	Total Fungal Concentration (CFU/m^3^)	Dominant Fungal Genera
1G	650	420	*Penicillium*, *Cladosporium*	440	490	*Cladosporium*
2G	1720	390	*Cladosporium*	420	730	*Cladosporium*
3G	310	350	*Cladosporium*	450	640	*Cladosporium*
4G	1570	450	*Cladosporium*	310	500	*Cladosporium*
5G	530	240	*Cladosporium*	420	630	*Cladosporium*
6G	2970	220	*Cladosporium*	290	750	*Cladosporium*
7G	530	250	*Cladosporium*	440	530	*Cladosporium*
8G	1640	510	*Penicillium*	560	300	*Cladosporium*
9G	5530	500	*Penicillium*, *Cladosporium*	340	360	*Cladosporium*
10G	1570	570	*Penicillium*, *Cladosporium*	570	490	*Cladosporium*
11G	590	1330	*Penicillium*, *Cladosporium*	1420	26,610	*Penicillium*
12G	600	840	*Penicillium*, *Cladosporium*	290	310	*Cladosporium*
13G	650	2290	*Penicillium*, *Cladosporium*	330	27,830	*Geotrichum*
14G	1500	800	*Penicillium*, *Cladosporium*	560	300	*Cladosporium*
15G	370	220	*Cladosporium*	400	480	*Cladosporium*
16G	510	590	*Cladosporium*	330	330	*Paecilomyces*

**Table 6:** Outdoor air samples, taken in Budapest during the reconstruction period. Samples marked with * are considered in-plume samples potentially affected by spores from the reconstruction site. Note that simulated dispersion sensitivities can only be interpreted relative to each other and should be multiplied with the (unestimated) spore emission rate to yield quantifiable concentrations. ERMI = Environmental Relative Moldiness Index.

Sampling Date	Distance and Direction from the Reconstruction Site	*Cladosporium* spp. CFU/m^3^	*Penicillium* spp. CFU/m^3^	ERMI	Simulated Dispersion Sensitivity log_10_ m^−3^
4 May 2022.	19 km E	890	0	–61	–5.0
17 May 2022.	4 km S	860	10	–51	–5.5
17 June 2022. *	2 km S	11,080	240	–47	–2.7
9 July 2022.	3 km W	2150	50	–50	<–7
19 July 2022. *	1 km SE	70	20	–32	–3.0
9 September 2022. *	2 km S	460	130	–49	–3.1
28 September 2022. *	2 km E	3770	100	–32	–3.0
26 October 2022. *	8 km NE	200	310	35	–2.8
28 October 2022.	16 km SE	1410	400	–18	–6.9
4 November 2022. *	2 km SW	4120	210	–46	–2.8
10 November 2022. *	1 km S	1140	100	–44	–2.5
12 November 2022.	8 km N	950	150	–48	<–7
15 November 2022.	9 km E	2630	120	–10	<–7
22 November 2022.	1 km S	630	40	–39	–6.9
22 November 2022.	3 km SW	970	80	10	<–7
25 November 2022.	11 km NE	180	140	–90	<–7
29 November 2022.	8 km S	990	10	–40	<–7
7 December 2022.	6 km E	90	20	–57	<–7
9 February 2023.	2 km E	40	30	–7	–5.6
24 February 2023. *	2 km SE	120	40	–30	–3.6

## Data Availability

Data are available upon request.

## Supplementary Materials

The following supporting information can be downloaded at: https://www.mdpi.com/article/10.3390/pathogens13121048/s1, Figure S1: Ratio of the detected fungal taxa during the 1^st^ sampling time of CRB; Figure S2: Ratio of the detected fungal taxa during the 2^nd^ sampling time of CRB; Figure S3: Ratio of the detected fungal taxa during the 3^rd^ sampling time of CRB; Figure S4: Ratio of the detected fungal taxa during after the 1^st^ disinfection (4^th^ sampling) of CRB; Figure S5: Ratio of the detected fungal taxa after the 2^nd^ disinfection (5^th^ sampling) of CRB; Figure S6: Ratio of the detected fungal taxa during the 1^st^ sampling time of FB; Figure S7: Ratio of the detected fungal taxa during the 2^nd^ sampling time of FB; Figure S8: Ratio of the detected fungal taxa during the 3^rd^ sampling time of FB; Figure S9: Ratio of the detected fungal taxa during after the 1^st^ disinfection (4^th^ sampling) of FB; Figure S10: Ratio of the detected fungal taxa after the 2^nd^ disinfection (5^th^ sampling) of FB; Figure S11: Ratio of the detected fungal taxa around the buildings after the 1^st^ disinfection (4^th^ sampling); Figure S12: Ratio of the detected fungal taxa around the buildings after the 2^nd^ disinfection (5^th^ sampling); Table S1: Environmental parameters during the whole sampling period. CRB = complete reconstruction building, FB = facadism building; Table S2: Different Aspergillus species in CRB. CRB = complete reconstruction building; Table S3: Different Aspergillus species in FB. FB = facadism building.
