# Supplementary material for: Indoor and Outdoor Air Microbial Contamination During Different Reconstruction Methods of Historic Buildings
Source: Pathogens. 2024 Nov 29;13(12):1048. doi: 10.3390/pathogens13121048 (PMC11728534; doi:10.3390/pathogens13121048)
Supplement: Supplementary file 1 [file pathogens-13-01048-s001.zip › pathogens-3291164-supplementary.pdf]

Figure S1. Ratio of the detected fungal taxa during the 1st sampling time of CRB.

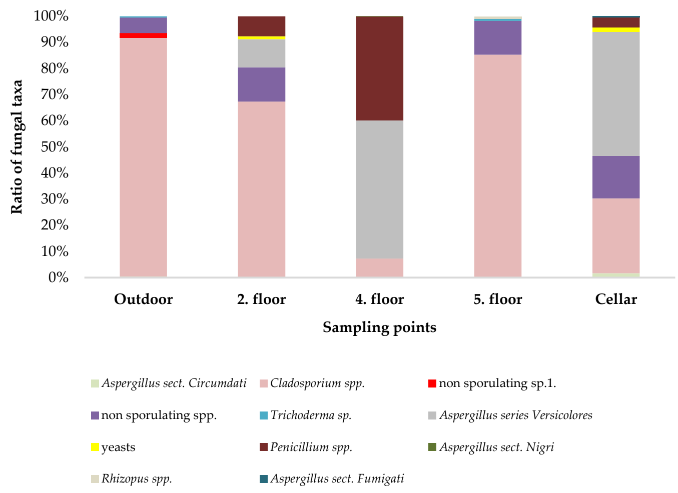

Figure S2. Ratio of the detected fungal taxa during the 2nd sampling time of CRB.

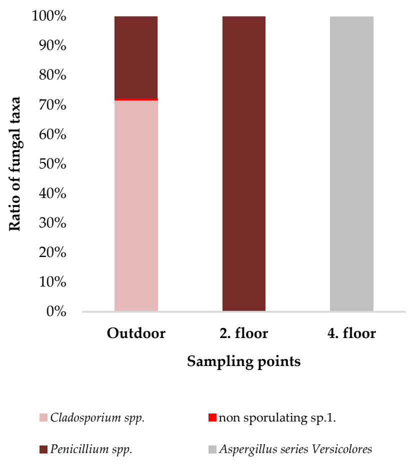

Figure S3. Ratio of the detected fungal taxa during the 3rd sampling time of CRB.

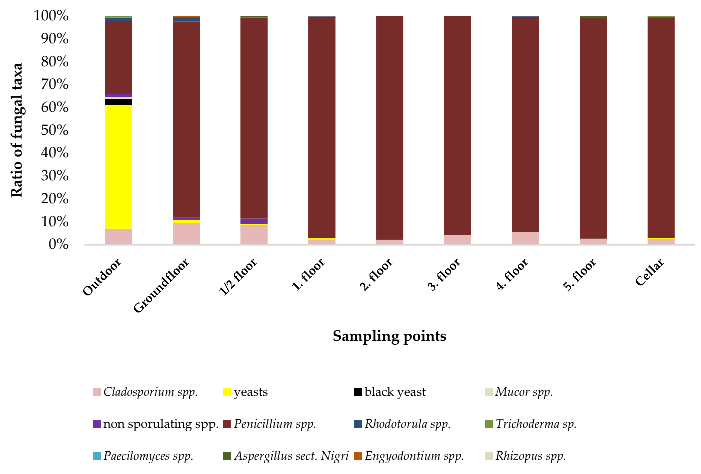

Figure S4. Ratio of the detected fungal taxa during after the 1st disinfection (4th sampling) of CRB.

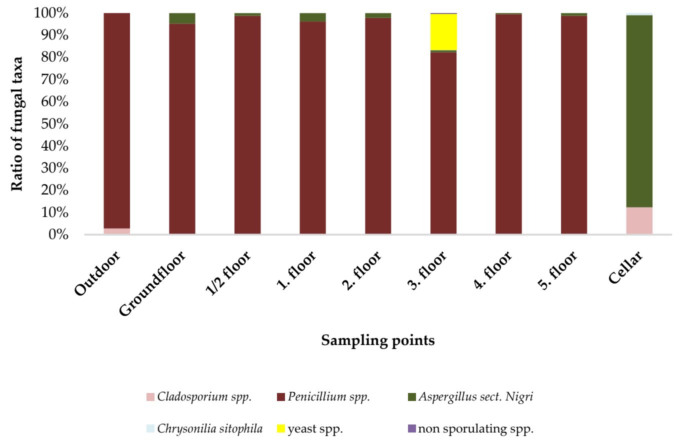

Figure S5. Ratio of the detected fungal taxa after the 2nd disinfection (5th sampling) of CRB.

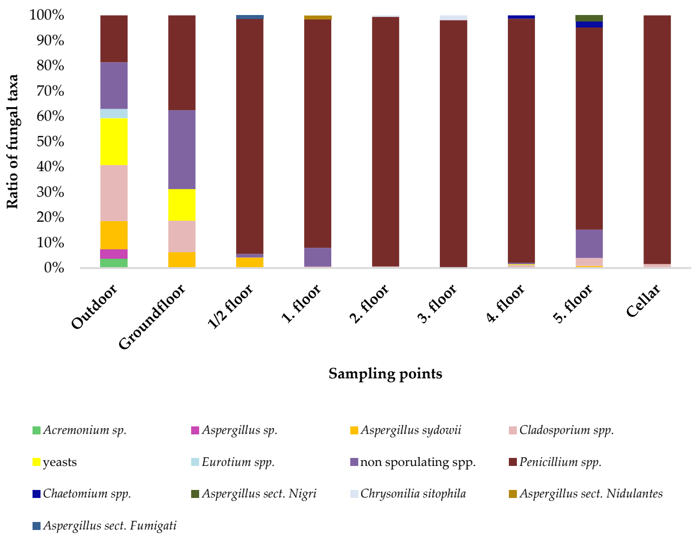

Figure S6. Ratio of the detected fungal taxa during the 1st sampling time of FB.

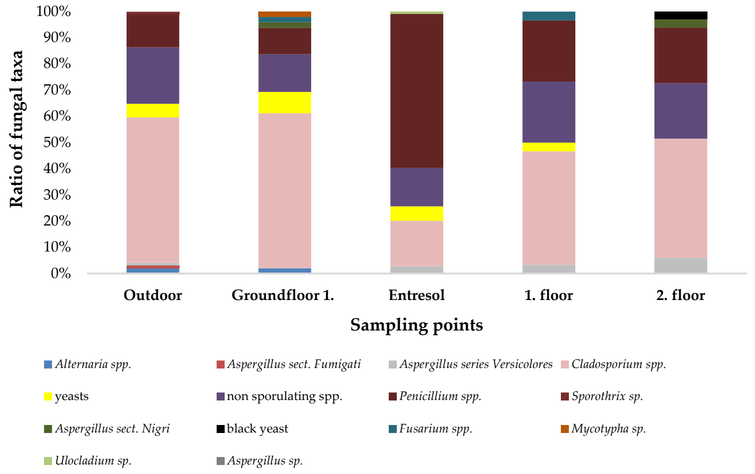

Figure S7. Ratio of the detected fungal taxa during the 2nd sampling time of FB

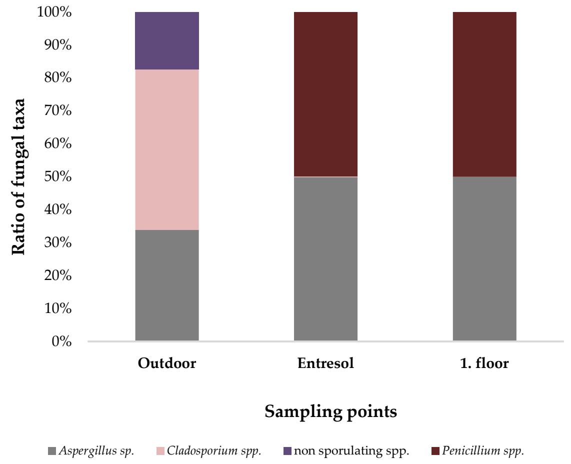

Figure S8. Ratio of the detected fungal taxa during the 3rd sampling time of FB.

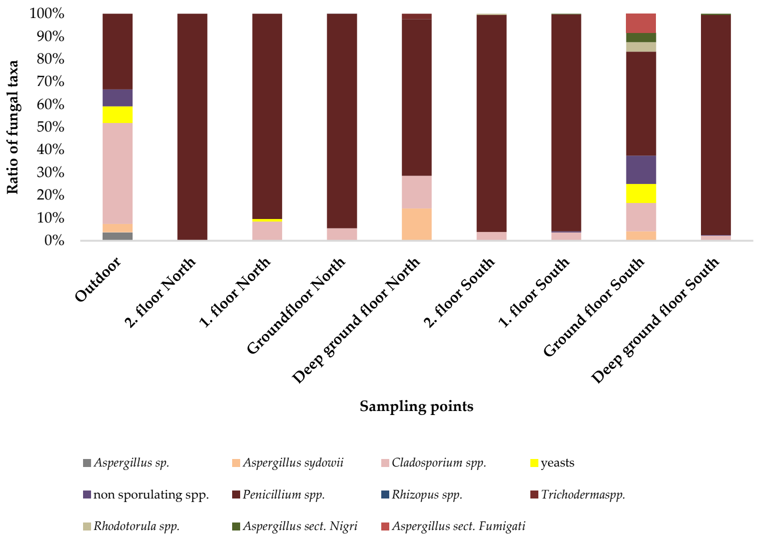

Figure S9. Ratio of the detected fungal taxa during after the 1st disinfection (4th sampling) of FB.

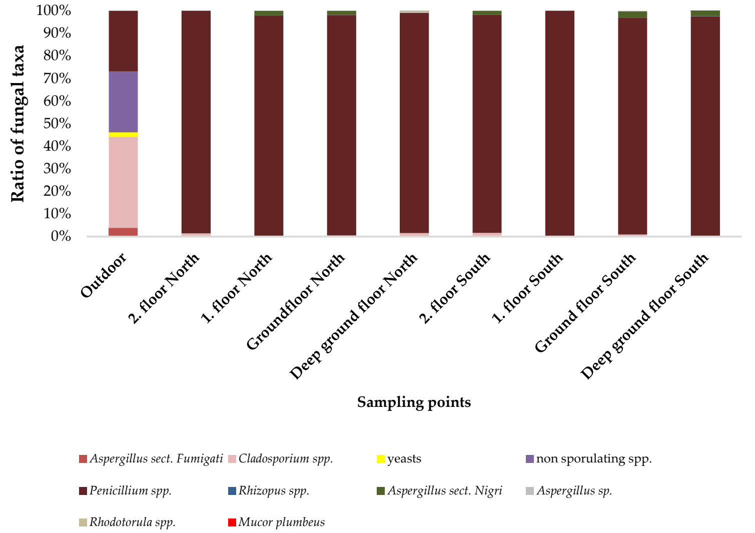

Figure S10. Ratio of the detected fungal taxa after the 2nd disinfection (5th sampling) of FB.

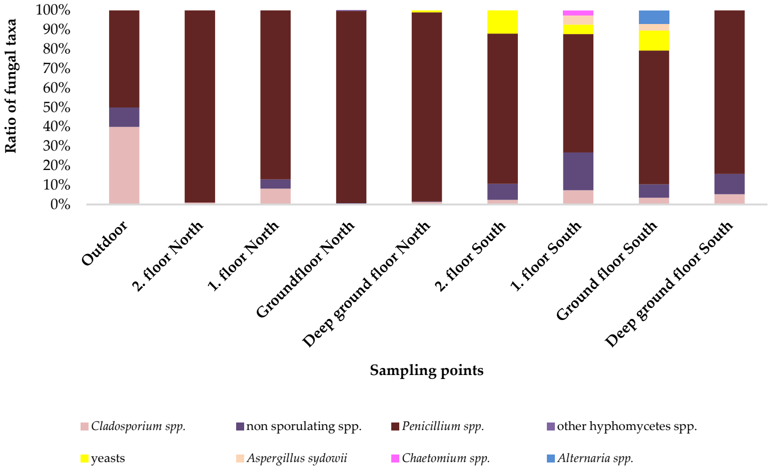

Figure S11. Ratio of the detected fungal taxa around the buildings after the 1st disinfection (4th sampling).

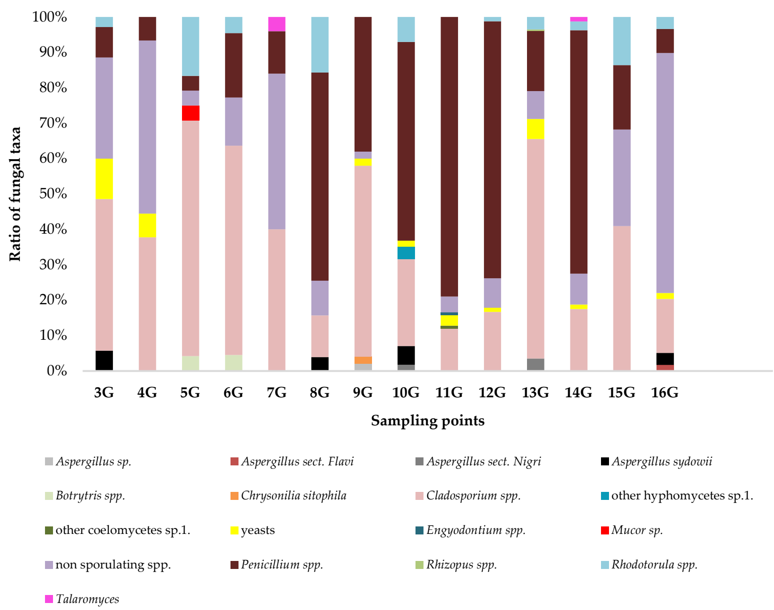

Figure S12. Ratio of the detected fungal taxa around the buildings after the 2nd disinfection (5th sampling).

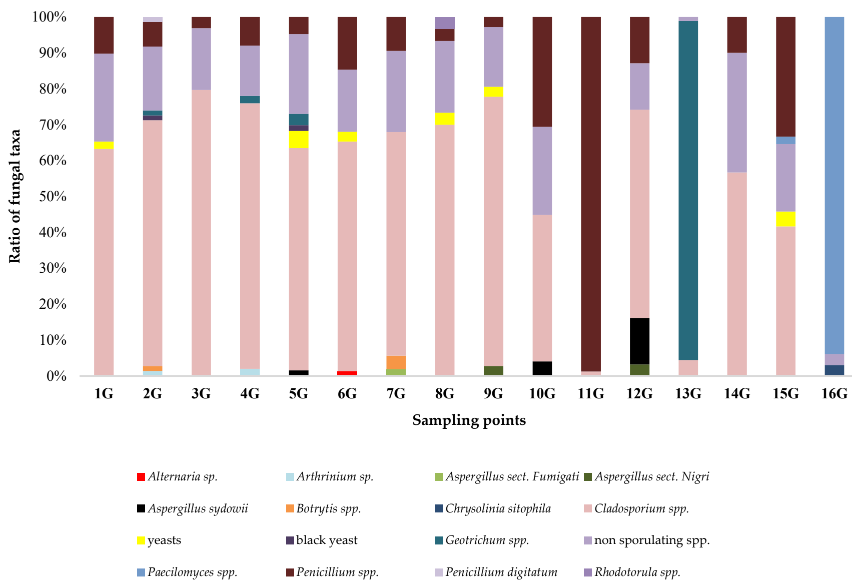

CRB			FB	
**Parameters**	**Outdoor 1.**	**Indoor 1.**	**Outdoor 1.**	**Indoor 1.**
**Relative humidity (%)**	45.2	52.1-67.8	24.6	45.5-53.3
**Temperature (°C)**	24.1	14.2-18.2	26.6	15.7-17.7
**Wind direction**	North-West	-	North	-
**Wind velocity (m/s)**	0.1	-	0.6	-
	**Outdoor 2.**	**Indoor 2.**	**Outdoor 2.**	**Indoor 2.**
**Relative humidity (%)**	54.4	66.0-67.5	51.4	53.3-65.0
**Temperature (°C)**	18.2	15.5-18.2	18.6	15.8-19.5
**Wind direction**	North-East	-	North-East	-
**Wind velocity (m/s)**	2.6	-	2.6	-
	**Outdoor 3.**	**Indoor 3.**	**Outdoor 3.**	**Indoor 3.**
**Relative humidity (%)**	63.7	40.3-56.7	47.8	48.0-62.9
**Temperature (°C)**	6.3	10.0-15.0	8.8	7.8-12.3
**Wind direction**	South-East	-	North-East	-
**Wind velocity (m/s)**	0.1	-	0.1	-
	**Outdoor 4.**	**Indoor 4.**	**Outdoor 4.**	**Indoor 4.**
**Relative humidity (%)**	81.0-95.0	62.2-76.1	81.0-95.0	69.8-82.6
**Temperature (°C)**	5.2-7.7	7.5-9.8	5.2-7.7	7.8-11.3
**Wind direction**	South-East	-	South-East	-
**Wind velocity (m/s)**	0.01	-	0.01	-
	**Outdoor 5.**	**Indoor 5.**	**Outdoor 5.**	**Indoor 5.**
**Relative humidity (%)**	50.6-65.2	58.2-81.5	50.6-65.2	65.6-85.4
**Temperature (°C)**	12.3-12.9	7.9-16.7	12.3-12.9	6.1-9.3
**Wind direction**	North	-	North	-
**Wind velocity (m/s)**	1.3	-	1.3	-

Table S1. Environmental parameters during the whole sampling period. CRB=complete reconstruction building, FB=facadism building.

<table>
<tr><th colspan="2">1st sampling</th><th colspan="2">2nd sampling</th><th colspan="2">3rd sampling</th><th colspan="2">4th sampling</th><th colspan="2">5th sampling</th></tr>
<tr><th>Outdoor I.</th><th>CRB I.</th><th>Outdoor II.</th><th>CRB II.</th><th>Outdoor III.</th><th>CRB III.</th><th>Outdoor IV.</th><th>CRB IV.</th><th>Outdoor V.</th><th>CRB V.</th></tr>
<tr><td rowspan="4">A. sect. Circumdati</td><td>A. sect. Circumdati</td><td rowspan="4">-</td><td rowspan="4">A. series Versicolores</td><td rowspan="4">-</td><td>A. sect. Nigri</td><td rowspan="4">-</td><td rowspan="4">A. sect. Nigri</td><td rowspan="4">Aspergillus sp.</td><td>A. sect. Nigri</td></tr>
<tr><td>A. series Versicolores</td><td rowspan="3">A. sydowii</td><td>A. sydowii</td></tr>
<tr><td>A. sect. Nigri</td><td>A. sect. Fumigati</td></tr>
<tr><td>A. sect. Fumigati</td><td>A. sect. Nidulantes</td></tr>
</table>

Table S2. Different *Aspergillus* species in CRB. CRB=complete reconstruction building.

<table>
<tr><th colspan="2">1st sampling</th><th colspan="2">2nd sampling</th><th colspan="2">3rd sampling</th><th colspan="2">4th sampling</th><th colspan="2">5th sampling</th></tr>
<tr><th>Outdoor I.</th><th>FB I.</th><th>Outdoor II.</th><th>FB II.</th><th>Outdoor III.</th><th>FB III.</th><th>Outdoor IV.</th><th>FB IV.</th><th>Outdoor V.</th><th>FB V.</th></tr>
<tr><td>A. sect. Fumigati</td><td>A. sect. Fumigati</td><td rowspan="4">Aspergillus sp.</td><td rowspan="4">Aspergillus sp.</td><td>Aspergillus sp.</td><td>Aspergillus sp.</td><td rowspan="4">A. sect. Fumigati</td><td>A. sect. Nigri</td><td rowspan="4">-</td><td rowspan="4">A. sydowii</td></tr>
<tr><td rowspan="3">A. series Versicolores</td><td>A. series Versicolores</td><td rowspan="3">A. sydowii</td><td>A. sydowii</td><td>A. sect. Fumigati</td></tr>
<tr><td>A. sect. Nigri</td><td>A. sect. Nigri</td><td rowspan="2">Aspergillus sp.</td></tr>
<tr><td>Aspergillus sp.</td><td>A. sect. Fumigati</td></tr>
</table>

Table S3. Different *Aspergillus* species in FB. FB=facadism building.
